# The Importance of a Genome-Wide Association Analysis in the Study of Alternative Splicing Mutations in Plants with a Special Focus on Maize

**DOI:** 10.3390/ijms23084201

**Published:** 2022-04-11

**Authors:** Zi-Chang Jia, Xue Yang, Xuan-Xuan Hou, Yong-Xin Nie, Jian Wu

**Affiliations:** 1 State Key Laboratory Breeding Base of Green Pesticide and Agricultural Bioengineering, Key Laboratory of Green Pesticide and Agricultural Bioengineering, Ministry of Education, Research and Development Center for Fine Chemicals, Guizhou University, Guiyang 550000, China; jiazc973@163.com; 2 State Key Laboratory of Crop Biology, College of Life Science, Shandong Agricultural University, Taian 271018, China; xueyang202001@163.com (X.Y.); 18854806295@163.com (X.-X.H.)

**Keywords:** alternative splicing, GWAS, QTL, proteogenomics, *Zea mays* L.

## Abstract

Alternative splicing is an important mechanism for regulating gene expressions at the post-transcriptional level. In eukaryotes, the genes are transcribed in the nucleus to produce pre-mRNAs and alternative splicing can splice a pre-mRNA to eventually form multiple different mature mRNAs, greatly increasing the number of genes and protein diversity. Alternative splicing is involved in the regulation of various plant life activities, especially the response of plants to abiotic stresses and is also an important process of plant growth and development. This review aims to clarify the usefulness of a genome-wide association analysis in the study of alternatively spliced variants by summarizing the application of alternative splicing, genome-wide association analyses and genome-wide association analyses in alternative splicing, as well as summarizing the related research progress.

## 1. Introduction

During gene expressions, a process may occur that results in the acquisition of different proteins starting from a single gene. This process, commonly known in biological sciences as alternative splicing (AS), occurs when certain exons are included or excluded from the final form of the mRNA produced by the gene. This process is an important way to achieve functional diversity of genes and explains that the existing diversity of the transcriptome and proteome exceeds the actual number of genes present in the genome of a given species [1]. Although its occurrence varies across the plant and animal kingdoms, it remains key to understanding growth, health and disease, as well as evolution and adaptations across species. Alternative splicing is involved in all stages of plant growth and development and plays an important role in seed development and flowering transition. Alternative splicing is also involved in the process of plant stress resistance, mainly playing a key role in abiotic stresses such as drought, extreme temperature and salinity.

At present, there are many methods to study alternative splicing variants: the RT-PCR method [2]; the expression sequence tag and cDNA sequence analysis method [3]; and single-molecule sequencing technology [4]. Genome-wide association analyses have been widely used in animal and plant research since they were first proposed. A population-level genome-wide association analysis has many advantages such as a wide detection range and high accuracy. Currently, in plants, a genome-wide association analysis is mainly used to locate genes or QTLs corresponding with a few complex traits. However, there are relatively few related studies on its alternative splicing in plants. The following is an overview of alternative splicing, genome-wide association analyses and the application of genome-wide association analyses in alternative splicing, aiming to highlight the application value of genome-wide association analyses in alternative splicing research.

## 2. Alternative Splicing: A Ubiquitous Regulatory Mechanism in Eukaryotes

### 2.1. Definition and Classification of Alternative Splicing

**Figure 1:**
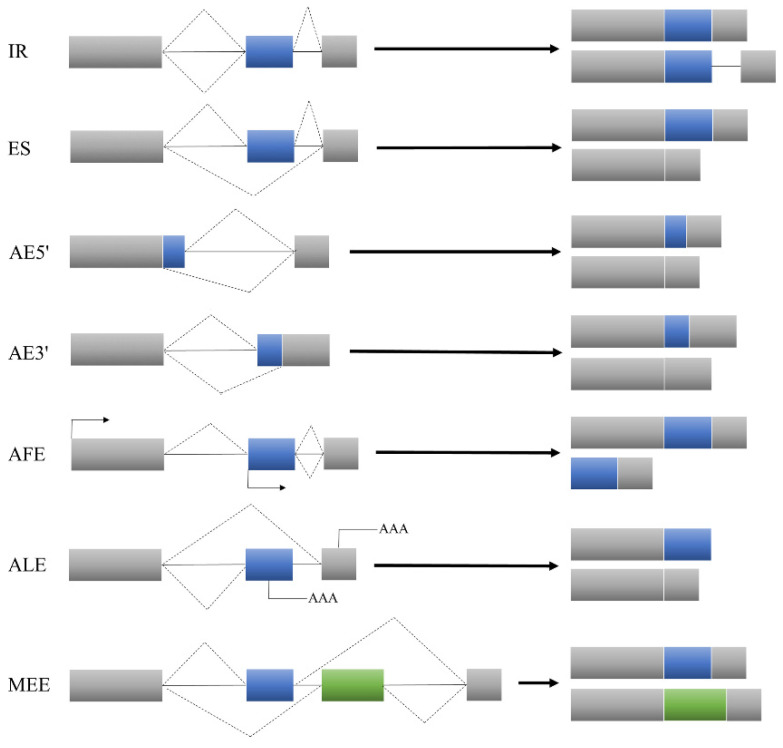
Alternative splicing species.

Alternative splicing is a regulatory mechanism that has been developed by organisms during evolution. It is a process by which pre-mRNA selects different splicing sites, excises introns and joins different exons together to generate multiple mature mRNA splicing isoforms [5,6]. A widespread natural variation in alternative splicing is observed in organisms, a process that increases the diversity of their transcriptomes and proteomes to improve survival. Alternative splicing events mainly include intron retention (IR), an alternative 5′ splice site (AE5′), an alternative first exon (AFE), exon skipping (ES), an alternative 3′ splice site (AE3′) and an alternative last exon (ALE). In addition, there is a more complex type called mutually exclusive alternative splicing of exons (MEE) (Figure 1) [7,8]. Alternative splicing is ubiquitous in eukaryotes. In plants, the predominant form of alternative splicing is intron retention [9,10,11]. In dicots, nearly 60% of *Arabidopsis* genes contain introns [12]. Among monocots, more than 70% of the intronic genes in rice give rise to different isoforms [11]. In maize, more than 50% of the genes are alternatively spliced; the genetic structure leading to a natural variation in alternative splicing is relatively simple and the cis-regulation effect is much higher than the trans-regulation effect [13]. This demonstrates that alternative splicing is essential for regulating the gene expression in plants.

### 2.2. Generation Mechanism of Alternative Splicing

**Figure 2:**
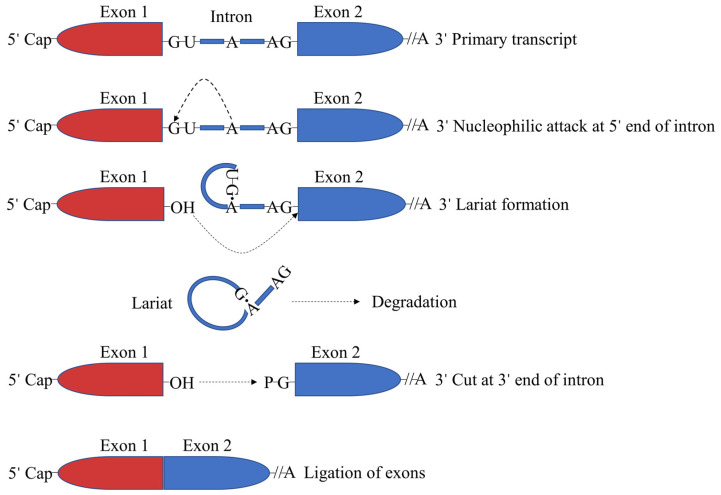
mRNA splicing mechanism.

The splicing process in organisms requires the participation of the spliceosome and its formation requires the complex synergy of various trans-acting factors. This includes the participation of more than 150 proteins such as snRNPs, U1, U2, U4/U6 and U5 [14]. The splicing process in the nucleus is mainly accomplished by two consecutive steps of transesterification. In the first transesterification reaction, the 5′ end of the intron is connected to the A base on the branch site to form a lariat structure. In the second transesterification reaction, the 3′ splice site is cut open, the left and right exons are connected by phosphodiester bonds and the intron is released in the form of a lariat and quickly degrades (Figure 2) [15,16,17]. Alternative splicing is primarily regulated by cis-acting elements and trans-acting factors.

RNA-binding proteins can regulate alternative splicing. For example, most S-serine, R-arginine (SR) proteins exist only in the nucleus, but a few can shuttle back and forth between the nucleus and cytoplasm. Typically, SR proteins primarily bind to exon splicing enhancers, promoting splicing by recruiting spliceosome proteins [18]. In contrast, heterogeneous nuclear ribonucleoprotein( hnRNP) family proteins antagonize the action of SR proteins by binding to exon splice silencers or intron splice silencers, thereby inhibiting splicing [9,19].

In addition, epigenetic factors regulate alternative splicing. Transcription and alternative splicing are not independent processes, as co-transcription (where RNA polymerase II acts as a link between transcription and splicing) can occur. The chromatin structure and histone modifications mainly affect RNA polymerase and splicing factors to regulate mRNA splicing. Furthermore, DNA methylation, the RNA secondary structure and non-coding RNAs also affect alternative splicing [9,16,20,21,22,23].

### 2.3. Output of Alternative Splicing

**Figure 3:**
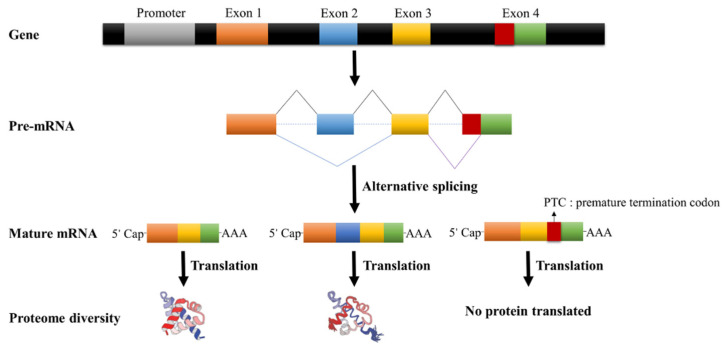
Alternative splicing process.

Importantly, alternative splicing increases the diversity of life [24,25]. Alternative splicing of genes can lead to the production of many isoforms, which indirectly leads to an increase in the diversity of proteins produced by translation. Furthermore, nonsense-mediated mRNA decay can affect alternative splicing by regulating mRNA stability [26,27,28]. In addition, alternative splicing competitively inhibits the function of transcription factors through a polypeptide interference mechanism and negatively regulates the expression of genes [29]. Finally, alternative splicing can also regulate the gene expression through miRNA, cutting and degrading the mRNA or blocking mRNA translation [30,31,32]. The detailed alternative splicing process in plants is shown in Figure 3.

### 2.4. Biological Function of Alternative Splicing in Plants

Alternative splicing is involved in the regulation of various plant life activities, especially the response of plants to abiotic stresses, and is also an important process of plant growth and development.

#### 2.4.1. Alternative Splicing Is Involved in the Regulation of Plant Growth and Development

Alternative splicing occurs in all stages of plant growth and development, mainly in the seed development stage and the flowering transition stage. *GRMZM2G104658* encodes a kinase of an unknown function that is alternatively spliced in three tissues of the seed, embryo and endosperm during the seed development, leading to its transition called a regulatory protein with a binding site that plays a role in seed development. NAC transcription factor 109 (*NACTF109*; *GRMZM2G014653*) is a regulator of abscisic acid, which is alternatively spliced during embryo development to regulate the ABA content in seeds and thus regulate seed dormancy. *LEAF RUST 10 DISEASE-RESISTANCE LOCUS RECEPTOR-LIKE PROTEIN KINASE-LIKE* (*LRK10L*, *GRMZM2G028568*) is alternatively spliced during the endosperm development, thereby assisting the ability of maize to regulate environmental stress during the developmental stages (Table 1) [33]. Pan et al. [34] showed that a reduced expression of *ZmSmk3* resulted in the defective splicing of mitochondrial nad4, resulting in mitochondrial damage, impaired embryonic and endosperm development and a smaller grain size. Xie et al. [35] analyzed the results of RNA sequencing of maize endosperm and identified 30 genes with intron retention that may be involved in maize endosperm development. Xiu et al. [36] found that EMP16 affected mitochondrial nad2 intron 4 splicing, thereby affecting embryogenesis and endosperm development in maize. Chen et al. [37] concluded that *ZmnMAT3* caused empty husks by regulating the splicing of mitochondrial group II introns during maize kernel development.

An important repressor of *Arabidopsis* flowering, *FLOWERING LOCUS C* (*FLC*), undergoes alternative splicing after vernalization [38]. The splicing factor AtU2AF65b is involved in the ABA-mediated regulation of the flowering time in *Arabidopsis* by splicing the *FLC* pre-mRNA [39]. As a splicing factor, SKI-interacting protein can affect the flowering time by regulating alternative splicing of splicing of early flowering (SEF) pre-mRNA in *Arabidopsis* [40].

#### 2.4.2. Alternative Splicing and Abiotic Stress in Plants

When the living environment changes, the property of plant sessile growth determines that plants must respond to environmental stress by adjusting their own physiological state. Alternative splicing plays an important role in this process. The abiotic stresses faced by plants include drought, extreme temperature and salinity. Abiotic stress can cause alternative splicing of related functional genes to produce gene products with different functions in plants, which are then used to combat abiotic stress.

Thatcher et al. [33] found that drought-induced AS in maize (*Zea mays* L.) mainly occurred in the leaves and ears; 1060 and 932 AS events were identified in those tissues, respectively. Under normal developmental conditions of the maize leaves, there were relatively few morphological changes and few differences in developmental splicing. However, under drought-treated conditions, the splicing events in the maize leaves increased. As the drought progressed, the splicing events gradually increased. This suggests that alternative splicing responds to abiotic stress. A transcriptome analysis of maize seeds, embryos and endosperm at different developmental stages revealed that *GRMZM2G104658* is an unknown kinase that is highly alternatively spliced and produces nine different known transcripts in three tissues. *GRMZM2G104658* reduces isoform 1 and increases isoform 7 in three tissue types the maize seed, embryo and endosperm resulting in a protein with regulatory functions. However, its role in seed development requires further investigation. *CORONATINE INSENSITIVE1* (*COI1*; *GRMZM2G353209*) is a jasmonic acid component involved in the stress response. Under drought conditions, COI1 produces isoforms containing Leu-rich repeat regions, resulting in an increased leaf sensitivity to jasmonic acid. *HSP93-V* (*GRMZM2G009443*) has increased Clp domain-containing transcripts under drought stress, which can increase protein degradation. Monodehydroascorbate reductase4 (*MDAR4*; *GRMZM2G320307*) encodes a monodehydroascorbate reductase that removes hydrogen peroxide. Under drought stress, MDAR4 increases hydrogen peroxide in response to stress through selective splicing. RNA-binding protein-defense related 1 (*BRN1*; *GRMZM2G005459*) regulates drought stress as a splicing factor. BRN1 undergoes alternative splicing under drought stress and participates in the splicing of other genes in response to stress. *U2AF65A* (*GRMZM5G813627*) is a U2 small nuclear ribonucleoprotein cofactor. *U2AF65A* undergoes drought stress with altered splicing patterns, encompassing isoforms of all three RNA recognition motifs (RRMs) in response to stress. The expression level of the splicing factor PRP18 (*GRMZM2G102711*) is closely related to alternative splicing in response to drought. Under well-watered conditions, the expression level of this gene was reduced. However, under drought conditions, the expression of this gene was greatly increased and the splicing of the ears and leaves significantly increased (Table 1). Tian et al. [41] demonstrated that the variable splicing of *ZmCCA1* was affected by the maize tissue type, as well as the photoperiod and drought stress. Alternative splicing of this gene mediated the response of the maize to heat and drought stress (Table 1). Li et al. [42] found that a high temperature enhanced alternative RNA splicing in maize and amplified the plant response to heat stress. An elevated temperature increased the frequency of major patterns of alternative splicing (AS) and particularly retained introns and skipped exons.

The *Arabidopsis* clock gene *CCA1* is alternatively spliced to generate CCA1β. CCA1β interferes with the formation of the dimers of *CCA1* and *LHY*, thereby inhibiting their transcription [43]. Yang et al. [44] demonstrated by methods such as RNA-seq that the *Arabidopsis* circadian clock not only controls the transcription of genes, but can also affect their post-transcriptional regulation by affecting alternative splicing. DREB2s in rice are dehydration response element (DRE)-binding proteins that regulate the expression of downstream genes involved in the drought response. Under normal growth conditions, *OsDREB2B* is mainly transcribed to produce *OsDREB2B1*. When rice is subjected to drought stress or exposed to a high temperature, OsDREB2B is mainly transcribed to produce OsDREB2B2, indicating that the resistance of rice to drought stress is affected by alternative splicing [45].

**Table 1:** Summary of splicing-associated genes [33,41].

Gene ID	Gene Name	Gene Function
*GRMZM2G353209*	*COI1*	Response to drought stress
*GRMZM2G009443*	*HSP93-V*	Heat shock protein
*GRMZM2G320307*	*MDAR4*	Removes hydrogen peroxide
*GRMZM2G005459*	*BRN1*	Salicylic acid responsive
*GRMZM5G813627*	*U2AF65A*	Splicing factor
*GRMZM2G104658*		Seed development
*GRMZM2G014653*	*NACTF109*	Abscisic acid (ABA) synthesis regulator
*GRMZM2G028568*	*LRK10L*	Endosperm development
	*CCA1* [41]	Drought response

Alternative splicing has been shown to play a role in a variety of plant processes including seed germination and the transition to flowering. Alternative splicing is also thought to play an important role in abiotic stress processes including drought, extreme temperature and salinity.

## 3. Overview of a Genome-Wide Association Analysis

### 3.1. Definition and Principles of a Genome-Wide Association Analysis

Due to the presence of a large number of SNPs in the genome, these variants are often used as molecular markers for a genome-wide association analysis (GWAS). A control analysis or correlation analysis at a genome-wide level can be achieved, which assists in the identification of genetic variations affecting complex traits. This method was first proposed in 1996 by Neil Risch and Kathleen Merikangas [46]. Klein et al. were the first to use this method to identify polymorphisms associated with age-related macular degeneration [47]. Since then, it has been applied to research on human diseases such as coronary heart disease [48], obesity [49] and type 2 diabetes [50]. In addition, an association analysis is widely used in plants as a popular method for understanding complex quantitative traits in natural populations.

There are two methods for the identification of plant quantitative trait loci (QTLs): a linkage analysis and an association analysis. Compared with a linkage analysis, which is based on a population construction, GWAS is based on a linkage disequilibrium (LD), which makes full use of the historical recombination accumulated during plant evolution to correlate genotypes and phenotypes. This increased recombination increases the genetic diversity of the population, which greatly improves the accuracy of the localization and reduces the tedious work of group building [51].

LD is a measure of the degree of correlation between two markers. Similar to chromosomal linkage, when two linked markers recombine, the linkage state between the two is broken and a linked or independent state appears in the population. This recombination contributes to the LD between the two markers [52,53]. The strength of the LD is closely associated with the accuracy of a GWAS. Generally, the breakdown of the LD across different species is used to accurately determine the gene positions within the species during the association analysis. For example, the attenuation distance of *Arabidopsis* is approximately 250 kb, that of rice is approximately 100 kb and that of maize is only approximately 1 kb, thus association analyses in maize can reach the gene level [52,54].

### 3.2. General Pipeline of a GWAS in Plants

**Figure 4:**
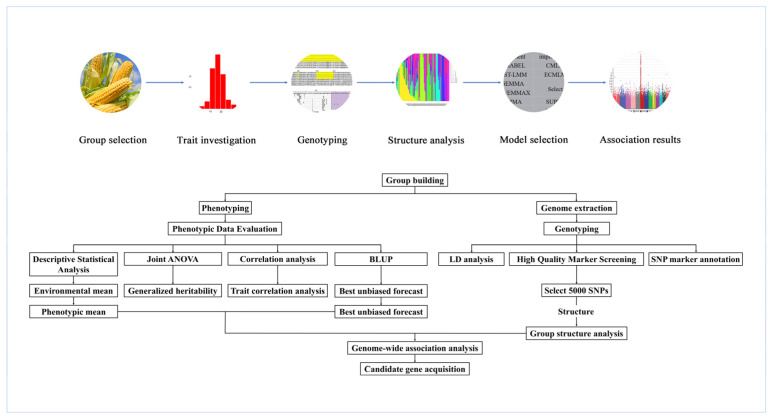
Schematic diagram of the GWAS process.

The process of GWAS research can be roughly divided into five steps (Figure 4). First, an appropriate association group is selected. A GWAS in crops can directly use natural variations within populations. When constructing a population, it is necessary to select materials with rich genetic variations, large morphological differences, broad genetic bases and regional representativeness. The sample size is generally approximately 300 to 500 individuals. Second, the phenotype of the target trait needs to be investigated. To reduce the influence of environmental and natural factors, the examination of phenotypes requires repeated experiments in multiple locations over many years. Third, the genotyping of the whole genomes of associated populations is performed using methods such as SNP arrays or resequencing. Fourth, the use of software such as STRUCTURE to analyze the population structure of the associated groups reduces the problem of high false-positive rates in the GWAS. Finally, analysis software such as TASSEL can be used to perform the GWAS on the genotypes and phenotypic traits of associated populations and to obtain loci that are significantly related to the phenotypic traits, which is conducive to the determination of subsequent candidate genes and the development of linked markers.

### 3.3. Progress of the GWAS in Maize Research

With the completion of the maize genome sequencing and the construction of multiple association groups, maize has become a model plant for association analyses. Maize has its own unique genetic characteristics and rich genetic diversity. In recent years, a GWAS has been widely used in the genetic analysis of various functional genes in maize. The following summarizes the application and research progress of the GWAS in maize in recent years.

There has been progress in the use of a GWAS in maize growth and development research. Liu et al. [55] genotyped 263 maize inbred lines using the SNP50 BeadChip maize array and confirmed that 4 SNPs were significantly associated with the starch content. Among the four candidate genes, *Glucose-1-phosphate* *adenylyltransferase* (*APS1*) was thought to be an important regulator of the starch content in the grain. Zheng et al. [56] performed a GWAS of 248 different maize inbred lines. It was found that the maize inbred lines showed an obvious natural variation regarding the grain quality under different environments. Further research identified a total of 29 genes related to the grain quality. Lu et al. [57] performed a GWAS of the total above-ground biomass and dry matter of different organs in 412 maize inbred lines. As a result, 1103 candidate genes were identified, after which a total of 224 genes detected by various GWAS models were considered to be of high confidence. Zhang et al. [58] studied the flowering traits in 310 maize inbred lines. Three flowering traits were mapped to SNP molecular markers through a GWAS. The results showed that there were 22 SNP markers associated with DTT (days to tasseling) and 234 candidate genes were identified near these SNP markers. Dong et al. [59] used an Illumina PE150 sequencer to perform the whole genome resequencing of 80 maize core inbred lines and obtained 1,490,007 SNPs for the subsequent association analysis. After two years of the GWAS, 10 SNP loci were significantly associated with the grain fat content. Six candidate genes were found in the linkage disequilibrium region of the most significant SNP site and may be closely related to the anabolism of fat.

In addition, a GWAS has been applied to the study of the maize stress response. Zhang et al. [60] studied the phenotypes associated with seed germination at a low temperature of 10 °C in 5 out of 300 maize inbred lines. Based on the FarmCPU model in the GWAS, 15 significant SNPs were identified that were associated with seed germination under cold stress and three of them were associated with multiple characteristics. Ma et al. [61] performed a GWAS on 305 maize inbred lines under salt stress and identified 7 significant SNPs; 120 genes were obtained by searching the LD regions of these loci. These 120 genes were analyzed by transcriptome data and *GRMZM2G333183* and *GRMZM2G075104* were identified as the key genes in response to salt stress. Xiang et al. [62] performed a GWAS on maize seedlings in arid environments and found 83 genetic variants that might be related to drought tolerance in seedlings. They then analyzed these variant loci and identified 42 candidate genes. The *ZmVPP1* gene, encoding vacuolar-type H^+^ pyrophosphatase, had the largest phenotypic contribution as indicated by the peak GWAS signal.

In summary, we found that the application of a GWAS in the genetic analysis of functional genes in maize could help us to obtain information regarding a large number of genes related to maize growth and development such as grain quality, grain fat synthesis, grain starch content, plant flowering traits and biomass. Several genes related to maize resistance were also found; for example, genes related to cold stress, salt stress and drought stress. The acquisition of these genes may help to improve the yield of maize from two aspects of plant growth and development, as well as providing an improvement in stress resistance.

## 4. Application of a GWAS in Alternative Splicing Studies

The expression of intracellular genes is inseparable from alternative splicing and is a key link in the central dogma of the transmission of genetic information from DNA to proteins. A differential splicing analysis describes the difference in the use of alternatively spliced transcripts between two or more samples. When the differential splicing analysis is performed at the population level, it can be used to assess the genetic factors involved in differential splicing changes. We refer to the sites of genetic variation that regulate the alternative splicing variants as splicing quantitative trait loci (sQTLs) [13]. A GWAS based on a linkage disequilibrium has been widely used to examine complex quantitative trait loci from the transcriptional level to the metabolic level and even to the level of complex agronomic traits and epigenetics. Researchers have analyzed splicing-level RNA-seq data using genome sequence and gene annotation information to identify the quantitative trait loci that control genetic variations at the genome-wide level.

With the development of sequencing technology and bioinformatics, researchers have used quantitative genetics to study the genetic variation of gene expressions. However, there are few reports on the genetic regulatory mechanism of plant alternative splicing variations. The following summarizes the progress of the GWAS in plant alternative splicing variation research and compares it with animal-related research to provide a reference for the application of a GWAS in plant alternative splicing variation research. It may contribute to the in-depth study of a GWAS in the study of plant alternative splicing.

In plant research, there are relatively few reports on AS using GWAS methods. In *Arabidopsis*, Yoo et al. [63] investigated sQTLs in 141 *Arabidopsis* individuals by a GWAS. The study identified 1694 candidate sQTLs. These candidate sQTLs were then interpreted using 107 trait-related published SNPs. As a result, 96 candidate sQTLs were identified that could explain the mechanism of trait-related SNPs. Among them, 25 sQTLs that affect the high differential expression of alternative splicing target exons between genotypes were further screened. They are distributed on different chromosomes and are associated with the growth, flowering and stress of *Arabidopsis* thaliana. Khokhar et al. [64], through a GWAS of 666 geographically distinct *Arabidopsis* ecotypes, found that trans-sQTLs were abundantly accumulated on chromosome 1. In addition, sQTLs co-localized with trait-associated SNPs were also identified. Among them, a large number of sQTLs were enriched in the genes that regulate the stress response, as well as flowering and the circadian clock, mediating the expression of multiple genes including circadian clock-associated 1 (*CCA1*), late elongated hypocotyl (*LHY*), phytochrome-interacting factor 5 (*PIF5*) and b-box domain protein 19 (*BBX19*). It indicated that the existence of differential isoforms is important in the regulation of different ecological types in *Arabidopsis*. An analysis of alternative splicing types also showed that although all types of AS contributed to sQTLs, intron retention (IR) was more prevalent than any other AS type. Yu et al. [65] identified a total of 764 genotype-specific splicing (GSS) events in rice–salt tolerance studies. Among them, five genes were significantly associated with the Na^+^ content. Ultimately, *OsNUC1* and *OsRAD23* emerged as the most likely candidate genes with splice variants. In studies of alternative splicing in maize, Mei et al. [66] analyzed AS differences between B73 and Mo17 via a linkage population. Chen et al. [13] used mixed linear models in a GWAS and combined 1.25 million SNPs to identify the sQTLs in 368 maize inbred lines. By performing an LD analysis of the associated SNPs in each gene detected by the sQTLs and examining and screening for changes in the splicing rate for each specific sQTL (rate of splicing difference exceeding 5%), a total of 19,554 unique sQTLs corresponding with 6570 genes were detected. The AS types involved in each sQTL were then analyzed. In total, 20,317 sQTL-related AS events were identified with IR being the most common (36%). In addition, the results of a functional enrichment analysis of the genes mapped to the sQTLs showed that the genes with alternative splicing variants mainly functioned in the cells and were involved in protein metabolism and RNA processing and binding in response to external environmental stimuli.

The use of a GWAS is more common in the study of alternative splicing in animals than in plants, especially in the study of human diseases. For example, existing GWAS studies have identified the existence of a large number of SNPs that are closely related to breast cancer (BC); these SNPs are mostly located in non-coding regions, suggesting a regulatory function. Machado et al. [67] identified BC-related SNPs through screening and the analysis of the genotype and expression data of breast tissue samples from the GTEx project and finally identified four sQTLs. Saharali et al. [68] performed RNA-seq on 376 whole blood samples from different individuals in the COPD (chronic obstructive pulmonary disease) Genetic Study. Through the GWAS linear model, 561,060 specific SNPs were identified and were closely related to 30,333 splice sites, which corresponded with 6419 genes. A GWAS has also been used to study various diseases including human multiple sclerosis [69], gliomas [70] and placental disorders [71]. The GWAS-related sQTLs are summarized in Table 2.

Combined with the research progress of the GWAS in maize in Section 2 and the study of the GWAS in plant alternative splicing variations in this section, it was found that the application of a GWAS in plants can be divided into two categories. First, a GWAS can be used to map several genes or QTLs corresponding with complex traits such as the mapping of plant growth, as well as development-related genes and stress resistance-related genes. Second, a GWAS can also be used to study alternatively spliced variants in plants. Through the detection and screening of the splicing ratio at the whole genome level, a large number of SNP variant sites related to alternative splicing have been found and these sites were associated with the corresponding traits. This contributes to our analysis of the possible regulatory mechanisms of alternative splicing to assess the contribution of the relevant phenotypic variation.

A comparison between the animal- and the plant-related studies revealed that the genetic structure of alternatively spliced variants in plants is simpler than in animal studies. In the regulation of splicing events, the proportion or priority of cis-acting and trans-acting factors detected by a GWAS is different in plants. For example, in *Arabidopsis*, the trans-acting elements are more abundant than the cis-acting elements [64]. However, in maize, alternative splicing is preferentially regulated by various cis-acting elements and cis-sQTLs explain more splicing variations than trans-sQTLs [13]. A GWAS is widely used in the study of alternative splicing in animals and has a relatively complete system. However, few studies in plants suggest that the application of a GWAS in the study of plant alternative splicing variants needs to be further explored. It may be possible to guide experimental research on plants with the help of existing research models in animals.

## 5. Conclusions and Prospects

Alternative splicing is common in eukaryotes. Relevant research advances in plants have shown that it plays a role in the growth and development of a variety of plants including seed germination and flowering. It also plays an important role in abiotic stresses such as drought, extreme temperature and salinity.

The application of a GWAS in the genetic analysis of the functional genes in maize showed that a GWAS can be used as a means of mapping genes or QTLs corresponding with complex traits; a large number of genes related to growth and development can be mapped through a GWAS such as grain quality, flowering and biomass, as well as the location of the genes related to stress responses (for example, salt stress, drought stress and low temperature stress). The acquisition of these genes helps to improve plants in terms of the plant growth process and stress resistance.

The application of a GWAS in plant alternative splicing can assist with quickly obtaining a large number of sQTLs in the whole genome of plants. These sQTLs with different traits can then be associated through identification and screening. The sQTLs associated with plant flowering, the circadian clock and other growth and development aspects distributed on different chromosomes, as well as the sQTLs associated with stress response, were obtained.

Due to advances in chip technology and sequencing technology, the cost of whole genome sequencing and gene chip technology should decrease and a GWAS may be more widely used in the study of alternative splicing variants. In addition, based on the application and development of proteomics such as SWATH-MS in plants [72,73], a GWAS may be combined with proteomics to further study the relationship between gene alternative splicing and trait regulations to discover the relevant regulatory networks to provide a theoretical basis for the treatment of animal diseases and increase crop yields.

## Figures and Tables

**Table 2:** Summary of GWAS-associated sQTLs.

SNP rsID	SNP Location	Gene	References
rs11249433	chr1	*SRGAP2D*	[67]
rs11552449	chr1	*DCLRE1B*	[67]
rs11552449	chr1	*PHTF1*	[67]
rs6504950	chr17	*STXBP4*	[67]
rs931794	chr15	*CHRNA5*	[68]
rs2202507	chr4	*GYPE*	[68]
rs10470936	chr4	*FAM13A*	[68]
rs2843128	chr1	*CDK11A*	[68]
rs4788084	chr16	*SULT1A2*	[68]
rs7740107	chr6	*L3MBTL3*	[69]
rs7572263	chr2	*C2orf80*	[70]
rs2699247	chr7	*SEC61G*	[70]
rs11216924	chr11	*TMEM25*	[70]
GRMZM2G041223	chr2	*ZmGRF8*	[13]
GRMZM2G171365	chr9	*ZmMADS1*	[13]
GRMZM2G348551	chr6	*sugary2*	[13]
GRMZM2G005887	chr1	*Cys2*	[13]
LOC_Os04g52960	chr4	*OsNUC1*	[65]
LOC_Os09g24200	chr9	*OsRAD23*	[65]

## Data Availability

Not applicable.

## References

[1] Verta J.P., Jacobs A. (2022). The role of alternative splicing in adaptation and evolution. Trends Ecol. Evol..

[2] Harvey S.E., Lyu J., Cheng C. (2021). Methods for Characterization of Alternative RNA Splicing. Methods Mol. Biol..

[3] Hirsch J., Lefort V., Vankersschaver M., Boualem A., Lucas A., Thermes C., d’Aubenton-Carafa Y., Crespi M. (2006). Characterization of 43 non-protein-coding mRNA genes in *Arabidopsis*, including the MIR162a-derived transcripts. Plant Physiol..

[4] Ding N., Cui H., Miao Y., Tang J., Cao Q., Luo Y. (2019). Single-molecule real-time sequencing identifies massive full-length cDNAs and alternative-splicing events that facilitate comparative and functional genomics study in the hexaploid crop sweet potato. PeerJ.

[5] Ellis J.D., Llères D., Denegri M., Lamond A.I., Cáceres J.F. (2008). Spatial mapping of splicing factor complexes involved in exon and intron definition. J. Cell Biol..

[6] Chen M.X., Zhang K.L., Gao B., Yang J.F., Tian Y., Das D., Fan T., Dai L., Hao G.F., Yang G.F. (2020). Phylogenetic comparison of 5′ splice site determination in central spliceosomal proteins of the U1–70K gene family, in response to developmental cues and stress conditions. Plant J..

[7] Chen M.X., Zhang K.L., Zhang M., Das D., Zhu F.Y. (2020). Alternative splicing and its regulatory role in woody plants. Tree Physiol..

[8] Fan T., Zhao Y.Z., Yang J.F., Liu Q.L., Tian Y., Debatosh D., Liu Y.G., Zhang J., Chen C., Chen M.X. (2021). Phylogenetic comparison and splice site conservation of eukaryotic U1 snRNP-specific U1–70K gene family. Sci Rep..

[9] Brown J., Kalyna M., Syed N.H., Marquez Y., Barta A. (2013). Alternative splicing in plants—Coming of age. Trends Plant Sci..

[10] Zhu F.Y., Chen M.X., Ye N.H., Shi L., Ma K.L., Yang J.F., Cao Y.Y., Zhang Y., Yoshida T., Fernie A.R. (2017). Proteogenomic analysis reveals alternative splicing and translation as part of the abscisic acid response in *Arabidopsis* seedlings. Plant J..

[11] Chen M.X., Mei L.C., Wang F., Boyagane Dewayalage I.K.W., Yang J.F., Dai L., Yang G.F., Gao B., Cheng C.L., Liu Y.G. (2021). PlantSPEAD: A web resource towards comparatively analysing stress-responsive expression of splicing-related proteins in plant. Plant Biotechnol. J..

[12] Zhou K. (2019). The alternative splicing of SKU5-Similar3 in *Arabidopsis*. Plant Signal Behav..

[13] Chen Q., Han Y., Liu H., Wang X., Sun J., Zhao B., Li W., Tian J., Liang Y., Yan J. (2018). Genome-Wide Association Analyses Reveal the Importance of Alternative Splicing in Diversifying Gene Function and Regulating Phenotypic Variation in Maize. Plant Cell.

[14] Ule J., Blencowe B.J. (2019). Alternative Splicing Regulatory Networks: Functions, Mechanisms, and Evolution. Mol Cell.

[15] Black D.L. (2003). Mechanisms of alternative pre-messenger RNA splicing. Annu. Rev Biochem..

[16] Hertel, Klemens J. (2014). Spliceosomal Pre-mRNA Splicing. Biol. Methods.

[17] Lee Y., Rio D.C. (2015). Mechanisms and Regulation of Alternative Pre-mRNA Splicing. Annu. Rev. Biochem..

[18] Jeong S. (2017). SR Proteins: Binders, Regulators, and Connectors of RNA. Mol. Cells.

[19] Geuens T., Bouhy D., Timmerman V. (2016). The hnRNP family: Insights into their role in health and disease. Hum. Genet..

[20] Brown S.J., Stoilov P., Xing Y. (2012). Chromatin and epigenetic regulation of pre-mRNA processing. Hum. Mol. Genet..

[21] Luco R.F., Allo M., Schor I.E., Kornblihtt A.R., Misteli T. (2011). Epigenetics in alternative pre-mRNA splicing. Cell.

[22] Naftelberg S., Schor I.E., Ast G., Kornblihtt A.R. (2015). Regulation of alternative splicing through coupling with transcription and chromatin structure. Annu. Rev Biochem..

[23] Masashi Y. (2017). Functions of long intergenic non-coding (linc) RNAs in plants. J. Plant Res..

[24] Severing E.I., Dijk A.D.J.V., Ham R.C.H.J.V. (2011). Assessing the contribution of alternative splicing to proteome diversity in *Arabidopsis* thaliana using proteomics data. BMC Plant Biol..

[25] Yang X., Coulombe-Huntington J., Kang S., Sheynkman G.M., Vidal M. (2016). Widespread Expansion of Protein Interaction Capabilities by Alternative Splicing. Cell.

[26] Chang Y.F., Imam J.S., Wilkinson M.F. (2007). The nonsense-mediated decay RNA surveillance pathway. Annu. Rev Biochem..

[27] Lykke-Andersen S., Jensen T.H. (2015). Nonsense-mediated mRNA decay: An intricate machinery that shapes transcriptomes. Nat. Rev. Mol. Cell Biol..

[28] Nasim Z., Fahim M., Hwang H., Susila H., Jin S., Youn G., Ahn J.H. (2021). Nonsense-mediated mRNA decay modulates *Arabidopsis* flowering time via the SET DOMAIN GROUP 40 – FLOWERING LOCUS C module. J. Exp. Bot..

[29] Seo P.J., Park M.J., Park C.M. (2013). Alternative splicing of transcription factors in plant responses to low temperature stress: Mechanisms and functions. Planta.

[30] Stepien A., Knop K., Dolata J., Taube M., Bajczyk M., Barciszewska-Pacak M., Pacak A., Jarmolowski A., Szweykowska-Kulinska Z. (2017). Posttranscriptional coordination of splicing and miRNA biogenesis in plants. Wiley Interdiscip. Rev. RNA.

[31] Pradillo M., Santos J.L. (2018). Genes involved in miRNA biogenesis affect meiosis and fertility. Chromosome Res..

[32] Li M., Yu B. (2021). Recent advances in the regulation of plant miRNA biogenesis. RNA Biol..

[33] Thatcher S.R., Danilevskaya O.N., Meng X., Beatty M., Zastrow-Hayes G., Harris C., Van Allen B., Habben J., Li B. (2016). Genome-Wide Analysis of Alternative Splicing during Development and Drought Stress in Maize. Plant Physiol.

[34] Pan Z., Ren X., Zhao H., Liu L., Tan Z., Qiu F. (2019). A Mitochondrial Transcription Termination Factor, ZmSmk3, Is Required for nad1 Intron4 and nad4 Intron1 Splicing and Kernel Development in Maize. G3.

[35] Xie S., Zhang X., Zhou Z., Li X., Huang Y., Zhang J., Weng J. (2018). Identification of genes alternatively spliced in developing maize endosperm. Plant Biol..

[36] Xiu Z., Sun F., Shen Y., Zhang X., Jiang R., Bonnard G., Zhang J., Tan B.C. (2016). EMPTY PERICARP16 is required for mitochondrial nad2 intron 4 cis-splicing, complex I assembly and seed development in maize. Plant J..

[37] Chen W., Cui Y., Wang Z., Chen R., He C., Liu Y., Du X., Liu Y., Fu J., Wang G. (2021). Nuclear-Encoded Maturase Protein 3 Is Required for the Splicing of Various Group II Introns in Mitochondria during Maize (*Zea mays* L.) Seed Development. Plant Cell Physiol..

[38] Sharma N., Geuten K., Giri B.S., Varma A. (2020). The molecular mechanism of vernalization in *Arabidopsis* and Cereals: Role of Flowering Locus C and its homologs. Physiol. Plant..

[39] Xiong F., Ren J.J., Yu Q., Wang Y.Y., Lu C.C., Kong L.J., Otegui M.S., Wang X.L. (2019). AtU2AF65b functions in abscisic acid mediated flowering via regulating the precursor messenger RNA splicing of ABI5 and FLC in *Arabidopsis*. New Phytol.

[40] Cui Z., Tong A., Huo Y., Yan Z., Yang W., Yang X., Wang X.X. (2017). SKIP controls flowering time via the alternative splicing of SEF pre-mRNA in *Arabidopsis*. BMC Biol..

[41] Tian L., Zhao X., Liu H., Ku L., Wang S., Han Z., Wu L., Shi Y., Song X., Chen Y. (2019). Alternative splicing of ZmCCA1 mediates drought response in tropical maize. PLoS ONE.

[42] Li Z., Tang J., Bassham D.C., Howell S.H. (2021). Daily temperature cycles promote alternative splicing of RNAs encoding SR45a, a splicing regulator in maize. Plant Physiol..

[43] Park M.J., Seo P.J., Park C.M. (2012). CCA1 alternative splicing as a way of linking the circadian clock to temperature response in *Arabidopsis*. Plant Signal. Behav..

[44] Yang Y., Li Y., Sancar A., Oztas O. (2020). The circadian clock shapes the *Arabidopsis* transcriptomeby regulating alternative splicing and alternative polyadenylation. J. Biol. Chem..

[45] Matsukura S., Mizoi J., Yoshida T., Todaka D., Ito Y., Maruyama K., Shinozaki K., Yamaguchi-Shinozaki K. (2010). Comprehensive analysis of rice DREB2-type genes that encode transcription factors involved in the expression of abiotic stress-responsive genes. Mol Genet. Genom..

[46] Risch N., Merikarigas K. (1996). The Future of Genetic Studies of Complex Human Diseases. Science.

[47] Klein R.J., Zeiss C., Chew E.Y., Tsai J.Y., Sackler R.S., Haynes C., Henning A.K., Sangiovanni J.P., Manne S.M., Mayne S.T. (2005). Complement factor H polymorphism in age-related macular degeneration. Science.

[48] Assimes T., Tcheandjieu C., Zhu X., Hilliard A., Huang J. (2021). A large-scale multi-ethnic genome-wide association study of coronary artery disease. Research square.

[49] Herbert A., Gerry N.P., McQueen M.B., Heid I.M., Pfeufer A., Illig T., Wichmann H.E., Meitinger T., Hunter D., Hu F.B. (2006). A common genetic variant is associated with adult and childhood obesity. Science.

[50] Saxena R., Voight B.F., Lyssenko V., Burtt N.P., de Bakker P.I., Chen H., Roix J.J., Kathiresan S., Hirschhorn J.N., Daly M.J. (2007). Genome-wide association analysis identifies loci for type 2 diabetes and triglyceride levels. Science.

[51] Flint-Garcia S.A., Thuillet A.C., Yu J., Pressoir G., Romero S.M., Mitchell S.E., Doebley J., Kresovich S., Goodman M.M., Buckler E.S. (2005). Maize association population: A high-resolution platform for quantitative trait locus dissection. Plant J..

[52] Flint-Garcia S.A., Thornsberry J.M., Buckler E.S., Thornsberry J.M., Buckler E.S. (2003). Structure of Linkage Disequilibrium in Plants. Annu. Rev. Plant Biol..

[53] Remington D.L., Thornsberry J.M., Matsuoka Y., Wilson L.M., Whitt S.R., Doebley J., Kresovich S., Goodman M.M., Buckler E.S.t. (2001). Structure of linkage disequilibrium and phenotypic associations in the maize genome. Proc. Natl. Acad. Sci. U S A.

[54] Kim S., Plagnol V., Hu T.T., Toomajian C., Clark R.M., Ossowski S., Ecker J.R., Weigel D., Nordborg M. (2007). Recombination and linkage disequilibrium in *Arabidopsis* thaliana. Nat. Genet..

[55] Liu N., Xue Y., Guo Z., Li W., Tang J. (2016). Genome-Wide Association Study Identifies Candidate Genes for Starch Content Regulation in Maize Kernels. Front. Plant Sci..

[56] Zheng Y., Yuan F., Huang Y., Zhao Y., Guo J. (2021). Genome-wide association studies of grain quality traits in maize. Sci. Rep..

[57] Lu X., Wang J., Wang Y., Wen W., Guo X. (2021). Genome-Wide Association Study of Maize Aboveground Dry Matter Accumulation at Seedling Stage. Front. Genet..

[58] Zhang H., Gao S., Binyang L.I., Zhong H., Zhang Z., Luo B. (2020). Genome-wide Association Analysis of Maize Flowering Traits. Asian Agric. Res..

[59] Dong Q.S., Dai L.Q., Lu W.U., Shi T.T., Wang Y.W., University J.A. (2018). Genome-wide Association Analysis of Fat Content in Maize. J. Maize Sci..

[60] Zhang Y., Liu P., Wang C., Zhang N., Shen Y. (2021). Genome-wide association study uncovers new genetic loci and candidate genes underlying seed chilling-germination in maize. PeerJ.

[61] Ma L., Zhang M., Chen J., Qing C., Shen Y. (2021). GWAS and WGCNA uncover hub genes controlling salt tolerance in maize (*Zea mays* L.) seedlings. Theor. Appl. Genet..

[62] Wang X., Wang H., Liu S., Ferjani A., Li J., Yan J., Yang X., Qin F. (2016). Genetic variation in ZmVPP1 contributes to drought tolerance in maize seedlings. Nat. Genet..

[63] Yoo W., Kyung S., Han S., Kim S. (2016). Investigation of Splicing Quantitative Trait Loci in *Arabidopsis* thaliana. Genom. Inf..

[64] Khokhar W., Hassan M.A., Reddy A.S.N., Chaudhary S., Jabre I., Byrne L.J., Syed N.H. (2019). Genome-Wide Identification of Splicing Quantitative Trait Loci (sQTLs) in Diverse Ecotypes of *Arabidopsis* thaliana. Front. Plant Sci.

[65] Yu H., Du Q., Campbell M., Yu B., Walia H., Zhang C. (2021). Genome-wide discovery of natural variation in pre-mRNA splicing and prioritising causal alternative splicing to salt stress response in rice. New Phytol.

[66] Mei W., Liu S., Schnable J.C., Yeh C.T., Springer N.M., Schnable P.S., Barbazuk W.B. (2017). A Comprehensive Analysis of Alternative Splicing in Paleopolyploid Maize. Front. Plant Sci..

[67] Machado J., Magno R., Xavier J.M., Maia A.T. (2019). Alternative splicing regulation by GWAS risk loci for breast cancer. BioRxiv.

[68] Saferali A., Yun J.H., Parker M.M., Sakornsakolpat P., Chase R.P., Lamb A., Hobbs B.D., Boezen M.H., Dai X., de Jong K. (2019). Analysis of genetically driven alternative splicing identifies FBXO38 as a novel COPD susceptibility gene. PLoS Genet..

[69] Alcina A., Fedetz M., Vidal-Cobo I., Andrés-León E., García-Sánchez M.-I., Barroso-del-Jesus A., Eichau S., Gil-Varea E., Luisa-Maria V., Saiz A. (2022). Identification of the genetic mechanism that associates L3MBTL3 to multiple sclerosis. Hum. Mol. Genet..

[70] Patro C.P.K., Nousome D., Lai R.K. (2021). Meta-Analyses of Splicing and Expression Quantitative Trait Loci Identified Susceptibility Genes of Glioma. Front. Genet..

[71] Ruano C.S.M., Apicella C., Jacques S., Gascoin G., Gaspar C., Miralles F., Méhats C., Vaiman D. (2021). Alternative splicing in normal and pathological human placentas is correlated to genetic variants. Hum Genet..

[72] Chen M.X., Zhang Y., Fernie A.R., Liu Y.G., Zhu F.Y. (2021). SWATH-MS-Based Proteomics: Strategies and Applications in Plants. Trends Biotechnol..

[73] Shen C.C., Chen M.X., Xiao T., Zhang C., Zhu F.Y. (2021). Global proteome response to Pb(II) toxicity in poplar using SWATH-MS-based quantitative proteomics investigation. Ecotoxicol. Environ. Saf..

